# Evaluation of diffuse myocardial fibrosis using contrast-enhanced look-locker cardiac MRI and its relation with cardiac function in dilated cardiomyopathy: comparison between 1.5T and 3T

**DOI:** 10.1186/1532-429X-14-S1-P152

**Published:** 2012-02-01

**Authors:** Masaki Tachi, Yasuo Amano, Shinichiro Kumita

**Affiliations:** 1 Radiology, Nippon Medical School, Tokyo, Japan

## Summary

The purpose of this study was to evaluate diffuse myocardial fibrosis by measuring myocardial and blood T1-values using contrast-enhanced Look-Locker cardiac MRI and comparing these values with left cardiac function in dilated cardiomyopathy (DCM). We also sought to assess the relation between the diffuse myocardial fibrosis and cardiac function on 1.5T and 3.0T.

## Background

Cardiac MRI is useful for evaluation of severity and prognosis of DCM.

This can render myocardial scarring in delayed contrast-enhanced MRI, but the scarring is observed only in 30%-40% of DCM cases presenting with congestive heart failure. Because of its binary contrast between the normal myocardium and scarring, delayed contras-enhanced MRI may fail to detect the diffuse myocardial fibrosis.

Some previous reports suggest that Look-Locker MRI at 1.5T can detect diffuse myocardial fibrosis, which has been observed by pathologic studies in DCM.

Though 1.5T imaging is widely used in clinical situations, 3.0T has potential to provide cardiac images with higher SNR and contrast. However, there is no report about the ability of 3.0T to detect the diffuse myocardial fibrosis in DCM.

## Methods

Thirty-three patients with DCM (1.5T: n = 17; 3.0T: n = 16) underwent contrast-enhanced cardiac MRI examination. Postcontrast myocardial T1-value was measured at the septum using Look-Locker imaging. The blood T1-value in the left ventricular chamber was also measured. The left ventricular cardiac functional parameters (i.e., EDV, ESV, LVEF, IVST) were measured using cine MRI. The correlations between the myocardial T1-value or (myocardial T1-value - blood T1-value) and the cardiac functional parameters noted above were evaluated both on 1.5T and 3.0T.

## Results

There were no significant differences in the left cardiac functional parameters between 1.5T and 3.0T groups. There was a significant inverse correlation between the myocardial T1-value and EDV (P<0.01, r= -0.71) or ESV (P<0.01, r= -0.69) on 1.5T.

The correlation was also found between the myocardial T1-value and ESV (P<0.05, r=-0.52), IVST (P<0.01 r=-0.52) or LVEF (P<0.01, r=-0.58) on 3.0T. However, no correlation was found between the myocardial T1 value and EDV on 3.0T (P = 0.39).

## Conclusions

Contrast-enhanced Look-Locker cardiac MRI may detect diffuse myocardial fibrosis related to left ventricular dysfunction in DCM on both 1.5T and 3.0T, by evaluating the reduced T1-value of the myocardium.

## Funding

We have not received funding.

**Figure 1:**
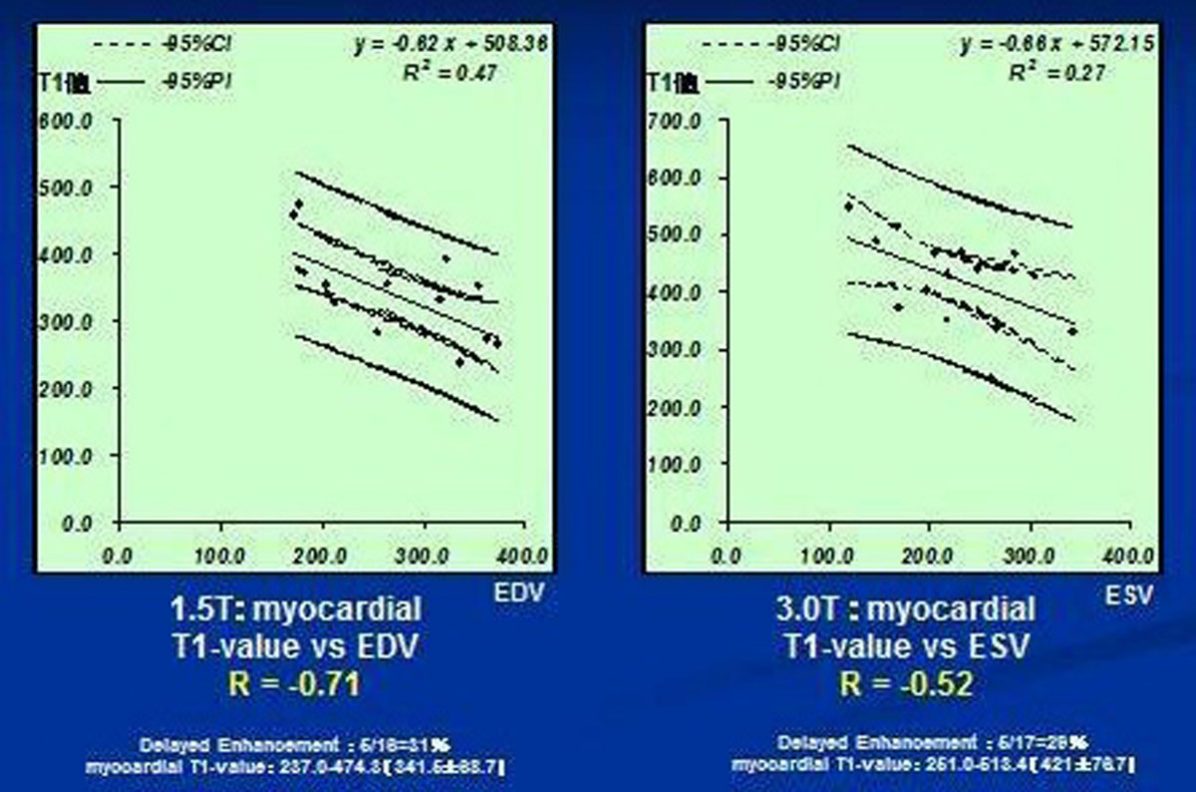
Contrast-enhanced Look-Locker cardiac MRI may detect diffuse myocardial fibrosis related to left ventricular dysfunction in DCM on both 1.5T and 3.0T.

